# 

**Published:** 1897-11

**Authors:** 


### CONTENTS.

COMMUNICATIONS.

The Study of Anatomy.......... 519
Phenomena of Cataphoresis.......... 525
Relations of Chemistry to Dentistry.......... 546
Orthopædia.......... 550
The Prominent Causes of Failure in the Use of Electricity in Dentistry.......... 553
The Facial Line and Angles in Prosthetic Dentistry.......... 556
Bridgework.......... 560
The Misapplication of Gold Crowns in Bridgework.......... 561
Continued Study of the Relations of the Frontal Sinus to the Antrum 562
The Libby Method.......... 563
Photo-Micography.......... 564

PROCEEDINGS.

Union of the American and Southern Dental Associations.......... 539

SELECTIONS.

Preserving Leeches.......... 545
Loss by Typhoid Fever.......... 564

NOTICES.

Indiana State Dental Association.......... 564

EDITORIAL

The Ohio State Dental Society.......... 565
What is in a Name?.......... 566
Bibliographical.......... 566

OBITUARY,

Dr. E. Magitot.......... 568

